# Ultra-early response assessment in lymphoma treatment: [^18^F]FDG PET/MR captures changes in glucose metabolism and cell density within the first 72 hours of treatment

**DOI:** 10.1007/s00259-018-3937-z

**Published:** 2018-02-26

**Authors:** Marius E. Mayerhoefer, Markus Raderer, Ulrich Jaeger, Philipp Staber, Barbara Kiesewetter, Daniela Senn, Ferdia A. Gallagher, Kevin Brindle, Edit Porpaczy, Michael Weber, Dominik Berzaczy, Ingrid Simonitsch-Klupp, Christian Sillaber, Cathrin Skrabs, Alexander Haug

**Affiliations:** 1 Department of Biomedical Imaging and Image-guided Therapy, Division of General and Pediatric Radiology, Medical University of Vienna, Waehringer Guertel 18-20, 1090 Vienna, Austria; 2 Department of Internal Medicine I, Medical University of Vienna, Vienna, Austria; 3 Department of Biomedical Imaging and Image-guided Therapy, Division of Nuclear Medicine, Medical University of Vienna, Waehringer Guertel 18-20, 1090 Vienna, Austria; 4 Cancer Research UK Cambridge Institute, University of Cambridge, Cambridge, UK; 5 Department of Radiology, Addenbrooke’s Hospital, Cambridge, UK; 6 Institute of Pathology, Medical University Vienna, Vienna, Austria

**Keywords:** Lymphoma, PET/MRI, FDG, Chemotherapy, Immunotherapy

## Abstract

**Purpose:**

To determine whether, in patients with Hodgkin lymphoma (HL) or non-Hodgkin lymphoma (NHL), [^18^F]FDG PET/MR can capture treatment effects within the first week after treatment initiation, and whether changes in glucose metabolism and cell density occur simultaneously.

**Methods:**

Patients with histologically proven HL or NHL were included in this prospective IRB-approved study. Patients underwent [^18^F]FDG PET/MR before, and then 48–72 h after (follow-up 1, FU-1) and 1 week after (FU-2) initiation of the first cycle of their respective standard chemotherapy (for HL) or immunochemotherapy (for NHL). Standardized [^18^F]FDG uptake values (SUVmax, SUVmean) and apparent diffusion coefficients (ADCmin, ADCmean) based on diffusion-weighted MRI, and metabolic and morphological tumour volumes (MTV, VOL) were assessed at each time-point. Multilevel analyses with an unstructured covariance matrix, and pair-wise post-hoc tests were used to test for significant changes in SUVs, ADCs, MTVs and VOLs between the three time-points.

**Results:**

A total of 58 patients (11 with HL and 47 with NHL) with 166 lesions were analysed. Lesion-based mean rates of change in SUVmax, SUVmean, ADCmin, ADCmean, MTV and VOL between baseline and FU-1 were −46.8%, −33.3%, +20.3%, +14%, −46% and −12.8%, respectively, and between baseline and FU-2 were −65.1%, −49%, +50.7%, +32.4%, −61.1% and −24.2%, respectively. These changes were statistically significant (*P* < 0.01) except for the change in VOL between baseline and FU-1 (*P* = 0.079).

**Conclusion:**

In lymphoma patients, [^18^F]FDG PET/MR can capture treatment-induced changes in glucose metabolism and cell density as early as 48–72 h after treatment initiation.

**Electronic supplementary material:**

The online version of this article (10.1007/s00259-018-3937-z) contains supplementary material, which is available to authorized users.

## Introduction

Positron emission tomography (PET) using the radiotracer [^18^F]FDG (2-[^18^F]fluoro-2-deoxy-d-glucose) is the current imaging technique of choice to assess treatment response in the majority of lymphoma subtypes [1–5]. It allows direct assessment of the glycolytic activity at the cellular level, which correlates with cell proliferation [6]. Diffusion-weighted imaging (DWI), an advanced magnetic resonance imaging (MRI) technique, can be used to assess tissue diffusivity which is an indirect measure of cell density. Since extracellular water movement in tumours is affected by cell density [7], DWI has recently been suggested as a potent alternative to [^18^F]FDG PET/CT to assess treatment response in lymphoma patients [8–12].

Previous studies have demonstrated that a reduction in glycolytic activity (i.e. [^18^F]FDG uptake on PET), and an increase in diffusivity (i.e. reduction of diffusion restriction on DWI, chiefly reflecting a reduction in cell density) can be observed after only a small number of therapy cycles [10, 12–15]. Notably, interim PET, which is typically performed after two to four therapy cycles and is included in the Lugano classification of the International Conference on Malignant Lymphoma (ICML) [1, 13], is currently under evaluation as a prognostic marker for different lymphoma subtypes, treatments and time-points, but so far with mixed results [15–22]. However, little is known about in vivo changes at the onset of therapy – in particular, to what extent do changes in glycolytic activity and cell density correlate with each other, and which method detects the earliest changes. In a murine lymphoma model, it has been shown that [^18^F]FDG PET can capture changes after treatment with etoposide as early as 16 h after treatment initiation [23]. So far, no comparable in vivo data in lymphoma patients are available. DWI has been shown to capture treatment response in lymphoma in vivo as early as 1 week after therapy initiation, but no earlier time-points were evaluated [24]. Finally, in a study in patients with Hodgkin lymphoma (HL) and diffuse large B-cell lymphoma (DLBCL), no significant changes in apparent diffusion coefficients (ADC) were observed approximately 2 days after therapy initiation, whereas [^18^F]FDG PET revealed treatment responses after 19 days [25].

Here, we investigated whether standard chemotherapy or immunochemotherapy led to significant changes in glucose metabolism, cell density, and lesion volume in lymphoma patients, at two time-points during the first week after treatment initiation. We particularly aimed to determine whether such ultra-early effects of treatment on glycolytic activity and cell density of lymphomas occur simultaneously, and whether they differ among histological subtypes, therapeutic regimens, and individuals with the same histology and treatment.

## Materials and methods

Patients with histologically proven, previously untreated malignant lymphoma who were referred to our institution for routine pretherapy [^18^F]FDG PET/MR were eligible for participation in our prospective exploratory study. Ethics committee approval and written informed consent from all patients were obtained prior to imaging. Inclusion criteria were the presence of one of the following histological lymphoma subtypes, as verified by a reference pathologist who analysed tissue samples (obtained by biopsy or during surgery) according to the current WHO classification of haematological and lymphoid malignancies, in combination with the following specified treatment schemes:

- Patients with HL scheduled for treatment with escalated BEACOPP
- Patients with DLBCL scheduled for treatment with either R-CHOP in those with an International Prognostic Index (IPI) of 1–3, or dose-adjusted (DA) EPOCH-R in those with an IPI >3 or double-hit lymphomas
- Patients with follicular lymphoma (FL) scheduled for treatment with R-BENDA
- Patients with mantle cell lymphoma (MCL) scheduled for treatment with R-BENDA

Patients with any of the following were excluded: women with a positive pregnancy test or clinically confirmed pregnancy; inability to understand the study goals or outline, or to consent to participation; age below the specified minimum of 18 years; known contraindication to MRI (e.g. implantable medical device according to the MRI Safety Guidelines, or conditions such as claustrophobia); or a blood glucose level >150 mg/dL. Patients who fulfilled the above requirements for participation underwent [^18^F]FDG PET/MR at three time-points:

1. Baseline: before treatment (with a specified range of 1–7 days before the first therapy cycle)
2. Follow-up 1 (FU-1): 48–72 h after initiation of the first therapy cycle
3. Follow-up 2 (FU-2): 1 week after initiation of the first therapy cycle

### Imaging protocols

All pretherapy and follow-up [^18^F]FDG PET/MR examinations were performed using a commercially available fully CE-certified integrated simultaneous PET/MR system (Biograph mMR; Siemens, Erlangen, Germany). This PET/MR system comprises a PET detector system inserted into a 3-T MRI system with high-performance gradient systems (45 mT/m) and a slew rate of 200 T/m/s. It is equipped with Total Imaging Matrix coil technology (Siemens) covering the body (from the vertex to the upper thighs) with multiple integrated radiofrequency surface coils that do not require repositioning. The PET system offers an axial field of view (FOV) of 258 mm, and a sensitivity of 13.2 cps/kBq.

[^18^F]FDG PET was performed 60 min after intravenous administration of a target dose of 3 MBq/kg (minimum injected dose 200 MBq) [^18^F]FDG with 5 min per bed position, three iterations and 21 subsets, a 4.2-mm slice thickness, and a 172 × 172 matrix, using the point spread function-based reconstruction algorithm HD-PET (Siemens). A coronal two-point Dixon T1-weighted VIBE 3D sequence was used for PET attenuation correction (i.e. separation into air, lungs, fat, and soft tissue) with a repetition time (TR) of 3.6 ms and echo times (TE) of 1.23 and 2.46 ms, one average, a 10° flip angle, a 79 × 192 matrix with a 328 × 500 mm FOV, and a 3-mm slice thickness with a 0.6-mm gap. An axial two-point Dixon T1-weighted VIBE 3D sequence was used for anatomic evaluation with a TR of 4.02 ms and TEs of 1.23 and 2.46 ms, a 10° flip angle, a 296 × 430 mm FOV and an image matrix of 154 × 320 with a 3-mm slice thickness with a 20% gap. In addition, an axial echoplanar DWI sequence with spectral adiabatic inversion recovery (SPAIR) was used during free-breathing for the entire anatomy, using b-values of 50 and 800, a TR of 6,800 ms and a TE of 63 ms, six averages and one echo, a 180° flip angle, a 168 × 104 matrix with a 440 × 340 mm FOV; and a 6-mm slice thickness with a 1.2-mm gap. ADC maps were generated automatically by the operational software supplied with the PET/MR scanner.

### Treatment protocols

Escalated BEACOPP was administered to patients with advanced stage HL and the presence of risk factors, according to the guidelines of the German and Austrian societies of haematology and oncology (DGHO and OeGHO; https://www.onkopedia.com). A 21-day cycle consisted of bleomycin 10 mg/m^2^ intravenously (i.v.) on day 8, etoposide 200 mg/m^2^ i.v. on days 1–3, doxorubicin 35 mg/m^2^ i.v. on day 1, cyclophosphamide 1,250 mg i.v. on day 1, vincristine 1.4 mg/m^2^ i.v. on day 8, procarbazine 100 mg/m^2^ orally (p.o.) on days 1–7, prednisone 100 mg/m^2^ p.o. on days 1–14. In these patients, FU-1 PET/MR was performed on day 3 following administration of the third BEACOPP dose.

In DLBCL patients, the choice between R-CHOP and DA-EPOCH-R treatments was based on each patient’s risk profile. High-risk patients with an IPI of >3 and patients with double-hit lymphomas were assigned to treatment with DA-EPOCH-R, whereas patients with an IPI of 1–3 were treated with R-CHOP. An R-CHOP cycle consisted of rituximab 375 mg/m^2^ i.v. on day 1, and cyclophosphamide 750 mg/m^2^ i.v., doxorubicin 50 mg/m^2^ i.v. and a vincristine 2 mg i.v. bolus on day 1 along with prednisone 50 mg/m^2^ p.o. on days 1–5. These patients had already received the full immunochemotherapy cycle (with the exception of prednisone, which was continued until day 5). In these patients, FU-1 PET/MR was performed on day 3. A DA-EPOCH-R cycle consisted of rituximab 375 mg/m^2^ i.v. on day 1, continuous infusion of etoposide 50 mg/m^2^, doxorubicin 10 mg/m^2^ and vincristine 0.4 mg/m^2^ on days 1–4, and cyclophosphamide 750 mg/m^2^ i.v. on day 5, along with 60 mg/m^2^ prednisone p.o. twice daily on days 1–5. In these patients, FU-1 PET/MR was performed on day 3 following administration of the third chemotherapy dose.

In patients with FL and MCL, an R-BENDA cycle consisted of rituximab 375 mg/m^2^ on day 1 and bendamustine 90 mg/m^2^ on days 2 and 3. In these patients, FU-1 PET/MR was performed on day 3 following administration of the second bendamustine dose.

### Image analysis

All [^18^F]FDG PET/MR examinations were evaluated by a board-certified radiologist and a board-certified nuclear medicine physician side-by-side to ensure that the same lesions were chosen for the quantitative analysis on [^18^F]FDG PET and DWI. The largest nodal or extranodal lymphoma manifestations of each involved anatomic region (with a specified maximum of five regions) that were visible on both [^18^F]FDG PET images (with a clear, focal, nonphysiological tracer accumulation) and DWI images (with a clear diffusion restriction, according to previously published criteria for malignancy [11]) were defined as target lesions on pretherapy [^18^F]FDG PET/MR, provided that they showed a long-axis lesion diameter of >1.0 cm for extranodal manifestations, or >1.5 cm for nodal manifestations on the T1-weighted or DWI images, as recommended in the Lugano/ICML guidelines [1]. Only for diffuse bone marrow involvement, for which histological verification prior to baseline imaging was required, could no size criteria be applied due to the lack of clear margins.

Maximum and mean standardized [^18^F]FDG uptake values (SUVmax, SUVmean) and metabolic tumour volumes (MTV, cm^3^) of these lesions were measured on the pretherapy (baseline)and the two follow-up (FU-1, FU-2) [^18^F]FDG PET/MR examinations, based on isocontour volumes of interest (VOIs) constructed using a 41% SUVmax threshold (as recommended by the European Association of Nuclear Medicine, and applied in previous studies [26–28]), using the *syngo* Multimodality Workplace environment (Siemens). Similarly, minimum and mean ADCs (ADCmin, ADCmean, ×10^−3^ mm^2^/s) of the target lesions were measured on pretherapy and follow-up ADC maps, based on manually defined three-dimensional VOIs, using the *syngo* environment (Siemens). Finally, manually defined VOIs were constructed on the T1-weighted images (guided, if necessary, by DWI), again using the *syngo* environment, to measure the morphological volumes (VOL, cm^3^) of the target lesions at baseline, FU-1 and FU-2. For focal bone and bone marrow lesions, which frequently remain visible as structural defects even in the absence of viable disease, MTVs were calculated, but not VOLs; whereas for diffuse bone marrow involvement, neither MTVs nor VOLs could be obtained due to the lack of clearly defined margins. For diffuse bone marrow involvement, SUVs and ADCs were extracted from spherical VOIs with a 1-cm diameter that were placed in pelvic bone marrow, avoiding the site of the biopsy, where reactive changes are known to occur.

If a lesion was no longer visible on the follow-up [^18^F]FDG PET images or ADC map, but was still visible as a residual mass/lesion on the respective T1-weighted MRI images, the latter were used for VOI definition and then copied. If a lesion was no longer visible on follow-up [^18^F]FDG PET images, the ADC map, or T1-weighted MRI images, SUVs, MTVs and VOLs were set to zero, whereas for ADCs for which no absolute maximum value existed, a spherical VOI with a 1-cm diameter was placed in the region where the lesion was observed at baseline taking care not to include areas of physiologically low ADCs (e.g. bowel contents).

### Statistical analysis

Mean rates of change in SUV, ADC, MTV and VOL between baseline and follow-up examinations (ΔSUVmax, ΔSUVmean, ΔADCmin, ΔADCmean, ΔMTV and ΔVOL) were determined for the target lesions, and also on a per-patient basis, using estimated marginal means of the patients’ target lesions. Lesion-based Pearson correlation coefficients (*r*) between ΔSUV and ΔADC were calculated. Multilevel analyses with an unstructured covariance matrix which showed the best model fit according to the Akaike information criterion were performed on a per-lesion basis; descriptive statistics were based on model estimates. Bonferroni-corrected post-hoc tests were used for pair-wise lesion-based comparisons of SUV, ADC, MTV and VOL among the three time-points (baseline, FU-1, and FU-2) based on pooled data from all lymphoma subgroups. Repeated-measures analysis of variance with post-hoc tests was used to compare baseline and FU-1 and FU-2 patient-based SUVs, ADCs, MTVs and VOLs using pooled data from all lymphoma subgroups. In the DLBCL subgroup, patient-based rates of change of the imaging parameters between baseline and FU-1 and between baseline and FU-2 were compared between the two treatment arms (R-CHOP vs. DA-EPOCH-R). The specified level of significance was *P* ≤ 0.05 for all tests. All statistical tests were performed using IBM SPSS Statistics 21.0 (IBM Corp., Armonk, NY).

## Results

Of 65 patients who fulfilled the inclusion criteria, seven were excluded at baseline due to elevated blood glucose levels (>150 mg/dL). Thus, a total of 58 patients (28 women and 30 men; mean age 58.9 ± 17.6 years, range, 25–92 years) fulfilled the criteria for participation in the study. Of these 58 patients, 11 were diagnosed with HL, 25 with DLBCL (15 in the R-CHOP arm, and 10 in the DA-EPOCH-R arm), 12 with FL, and 10 with MCL. One patient with FL and one patient with MCL underwent [^18^F]FDG PET/MR at baseline and FU-1, but not at FU-2; whereas one patient with DLBCL (R-CHOP arm) underwent [^18^F]FDG PET/MR at baseline and FU-2, but not at FU-1. A total of 166 target lesions (134 nodal, 32 extranodal; Table 1) were analysed.

**Table 1:** Absolute numbers of analysed target lesions by anatomic region

Region	HL	DLBCL	FL	MCL	Total
Nodal					
Cervical	1	2	2	4	9
Infraclavicular	4	2	0	0	6
Axillary	0	6	1	1	8
Mediastinal	14	4	2	2	22
Hilar	8	2	0	1	11
Mesenteric	2	8	2	3	15
Periaortic	1	8	4	4	17
Pelvic	0	8	13	8	29
Inguinal	0	5	8	4	17
Extranodal					
Waldeyer’s ring	0	1	0	0	1
Lungs	0	1	0	4	5
Spleen	1	0	0	0	1
Stomach	0	2	0	1	3
Small intestine	0	1	0	0	1
Large intestine	0	1	0	0	1
Bones	1	6	5	0	12
Soft tissues	0	2	0	0	2
Other	0	5	1	0	6

*HL* Hodgkin lymphoma, *DLBCL* diffuse large B-cell lymphoma. *FL* follicular lymphoma, *MCL* mantle cell lymphoma

**Fig. 1:**
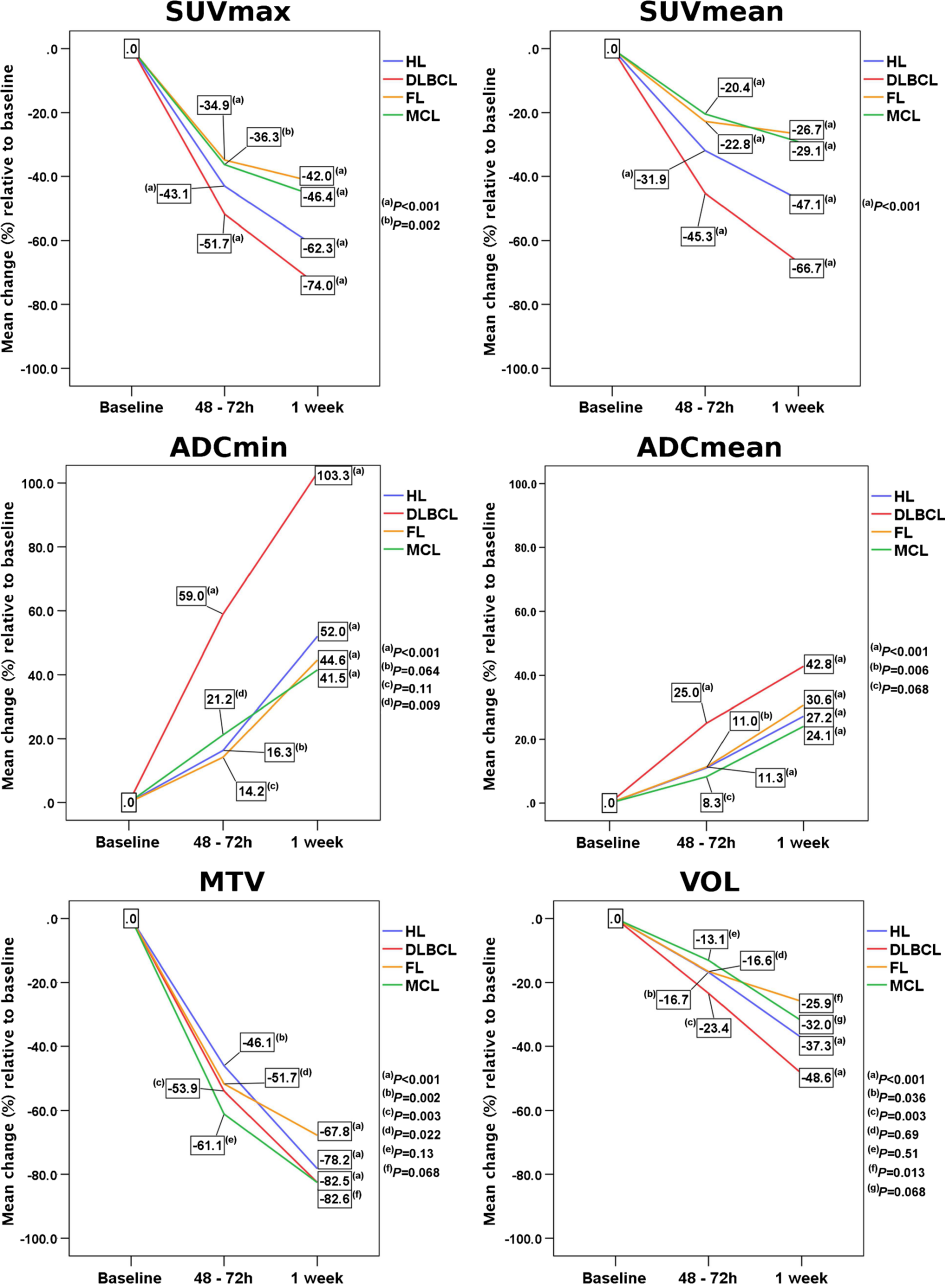
Lesion-based rates of change of standardized [^18^F]FDG uptake values (SUVmax, SUVmean), apparent diffusion coefficients (ADCmin, ADCmean), metabolic tumour volumes (MTV) and morphological tumour volumes (VOL) between baseline and 1 week separately for the four histological lymphoma subtypes

**Fig. 2:**
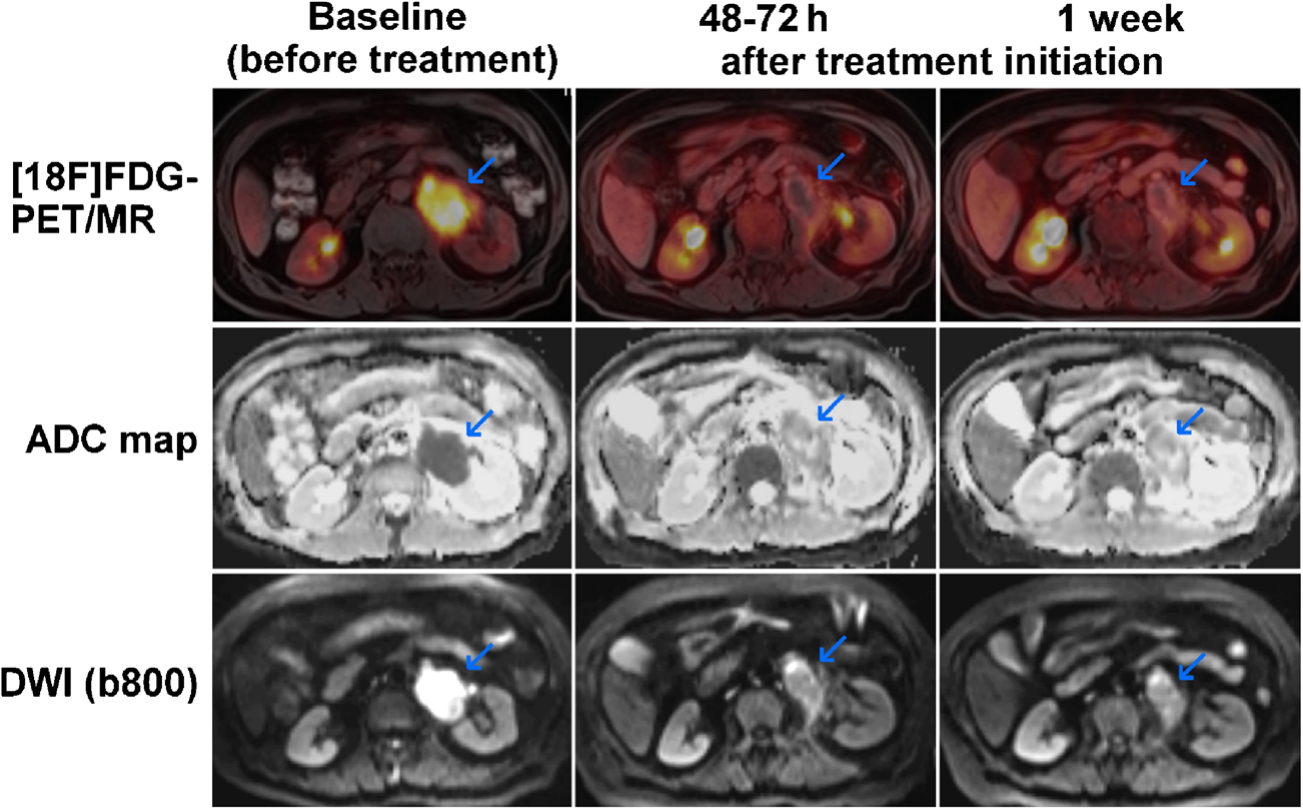
A 68-year-old woman with DLBCL of the left adrenal gland (*blue arrows*). The lymphoma shows intense glucose metabolism (high [^18^F]FDG uptake; high SUVs) at baseline on the axial fused [^18^F]FDG PET/MR image, and a clear diffusion restriction on the DWI image (high signal) and the ADC map (low signal). The PET/MR image at 48–72 h after treatment initiation (R-CHOP) shows an almost complete reduction in [^18^F]FDG uptake (reduced glucose metabolism) and a clear increase in tissue diffusivity (increased ADC, indicating reduced cell density) at this time-point. The PET/MR image at 1 week after treatment initiation shows an additional moderate decrease in lesion size relative to the baseline image

On a per-lesion basis, pooled SUVmax, SUVmean, ADCmin and ADCmean values from all lymphoma subtypes differed significantly between baseline and FU-1 (−46.8%, −33.3%, +20.3% and +14%, respectively; *P* < 0.001), between baseline and FU-2 (−65.1%, −49%, +50.7% and +32.4%, respectively; *P* < 0.001), and between FU-1 and FU-2 (−34.5%, −23.5%, +24.2% and +15.2%, respectively; *P* < 0.001). MTV also differed significantly between baseline and FU-1 (−46%; *P* = 0.002), and between baseline and FU-2 (−61.1%; *P* < 0.001), but not between FU-1 and FU-2 (−28%; *P* = 0.79) whereas VOL differed significantly only between baseline and FU-2 (−24.2%; *P* < 0.001), and not between baseline and FU-1 (−12.8%; *P* = 0.079), or between FU-1 and FU-2 (−13.1%; *P* = 0.154; Table 2, Figs. 1 and 2).

**Table 2:** Lesion-based pooled arithmetic means and 95% confidence intervals of standardized uptake values (SUV), apparent diffusion coefficients (ADC), metabolic tumour volumes (MTV), and morphological volumes (VOL) before (baseline), 48–72 h after, and 1 week after treatment initiation

	Baseline	48–72 h	1 week
SUVmax	10.9 (10.1–11.8)	5.8 (5.0–6.7)	3.8 (3.0–4.7)
SUVmean	5.1 (0–13.8)	3.4 (0–12.1)	2.6 (0–11.0)
ADCmin (×10^−3^ mm^2^/s)	518.6 (409.5–627.7)	629.2 (520.1–738.4)	781.7 (672.4–891.0)
ADCmean (×10^−3^ mm^2^/s)	925.0 (898.9–971.1)	1,054.6 (1,018.5–1,090.8)	1,215.2 (1,178.6–1,251.7)
MTV (cm^3^)	75.4 (0–438.4)	40.7 (0–395.2)	29.3 (0–343.8)
VOL (cm^3^)	103.7 (0–422.2)	90.4 (0–408.9)	78.6 (0–397.1)

On a per-patient basis, pooled SUVmax, SUVmean, ADCmin and ADCmean values from all lymphoma subtypes differed significantly between baseline and FU-1 (−54.9%, −41.7%, +27.8% and +17.8%, respectively; *P* < 0.001), and between baseline and FU-2 (−70.7%, −55.9%, +58% and +34.4%, respectively; *P* < 0.001). SUVmean, ADCmin and ADCmean, but not SUVmax, differed significantly between FU-1 and FU-2 (−24.6%, +23.6%, +14.1% and −35%, respectively; *P* = 0.015, *P* < 0.001, *P* < 0.001 and *P* = 0.094). MTV also differed significantly between baseline and FU-1 (−42.9%; *P* = 0.01), and between baseline and FU-2 (−53.9%; *P* = 0.001), but not between FU-1 and FU-2 (−19.2%; *P* = 1.0), whereas VOL differed significantly only between baseline and FU-2 (−18.7%; *P* = 0.002), and not between baseline and FU-1 (−12%; *P* = 0.069), or between FU-1 and FU-2 (−7.6%; *P* = 0.62). Line graphs revealed major differences in treatment response between individual patients, and even between patients with the same lymphoma subtype and treatment regimen (Online Resource 1).

In the DLBCL group, all patient-based imaging parameter changes from baseline to FU-1 except for ADCmin differed significantly between the two treatment arms (R-CHOP vs. DA-EPOCH-R), whereas only changes in SUVmean and VOL between baseline and FU-2 differed significantly between the two treatment arms (Table 3, Online Resource 2).

**Table 3:** Patient-based mean rates of change (%) of the six imaging parameters relative to baseline for the two DLBCL treatment arms (R-CHOP, DA-EPOCH-R)

	R-CHOP	DA-EPOCH-R	*P* value
ΔSUVmax			
48–72 h	−69.9 ± 27.9	−41.1 ± 17.2	0.005
1 week	−78.7 ± 23.2	−67.7 ± 18.4	0.20
ΔSUVmean			
48–72 h	−65.4 ± 23.6	−34.0 ± 13.2	<0.001
1 week	−74.8 ± 24.4	−57.0 ± 21.3	0.007
ΔADCmin			
48–72 h	65.9 ± 71.9	50.7 ± 49.9	0.55
1 week	103.8 ± 83.3	93.5 ± 79.0	0.76
ΔADCmean			
48–72 h	41.8 ± 28.7	17.0 ± 18.0	0.016
1 week	57.9 ± 32.7	35.4 ± 23.9	0.059
ΔMTV			
48–72 h	−73.2 ± 30.5	−41.2 ± 32.1	0.023
1 week	−84.6 ± 19.8	−75.5 ± 18.3	0.25
ΔVOL			
48–72 h	−38.1 ± 28.6	−7.9 ± 17.3	0.005
1 week	−60.2 ± 24.0	−30.2 ± 24.2	0.009

Weak, statistically significant negative correlations were observed between lesion-based ΔSUVmax and ΔADCmin (baseline to FU-1 *r* = −0.32, *P* < 0.001; baseline to FU-2 *r* = −0.32, *P* < 0.001), whereas moderate significant negative correlations were observed between ΔSUVmean and ΔADCmean (baseline to FU-1 *r* = −0.54, *P* < 0.001; baseline to FU-2 *r* = −0.41, *P* < 0.001).

## Discussion

The observed decrease in lymphoma glucose metabolism (i.e. FDG uptake, measured in terms of SUV and MTV) and increase in tissue diffusivity (i.e. ADC, chiefly reflecting cell density) in our study clearly suggest the presence of treatment effects, which can be measured in vivo by [^18^F]FDG PET/MR as early as 48–72 h after treatment initiation. Further imaging parameter changes reflecting treatment effects clearly occurred during the first week. Notably, significant changes during the first week after treatment initiation were found not only in SUVs (and the associated MTVs) and ADCs, but also in morphological volumes, albeit to a lesser degree.

A closer look at the rates of change of the imaging parameters clearly suggests that, regardless of histological subtype, glucose metabolism-based measures (SUV and MTV) show a more pronounced response to treatment during the first 48–72 h after treatment initiation, whereas the changes between 72 h and 1 week appeared to be less pronounced. For diffusivity/cell density, as reflected by ADCs, the opposite effect was generally observed, with a less pronounced response to treatment during the first 48–72 h after treatment initiation, and a more pronounced response between 72 h and 1 week; only DLBCL, in which changes in ADC over time more closely resembled those in SUV, differed in this regard. Significant changes in morphological lesion volumes were also observed at these early time-points, although they were only small-to-moderate, particularly during the first 48–72 h after treatment initiation, when, contrary to changes after 1 week, they did not reach statistical significance. This very early reduction in lesion size under treatment confirms the results of Horger et al. [24], who found a significant reduction in lesion size in a small sample of responding lymphoma patients 1 week after treatment initiation. The fact that, in the latter study, lesion shrinkage was less pronounced than in the present study may possibly be explained by the fact that transaxial lesion diameters, instead of volumes, were used, and also the study population of Horger et al. included only three patients with DLBCL, which was the subtype that showed by far the largest volume decreases in our study.

Clear differences in treatment responses and associated changes in imaging parameters were seen among the four histological lymphoma subtypes in our study. DLBCL showed the largest changes, followed by HL, followed by MCL, with FL showing the smallest changes. On the other hand, only patients with FL and MCL received the same treatment regimen, whereas treatment regimens differed among those with DLBCL and HL, and hence the latter two subtypes cannot be directly compared with each other, or with FL and MCL. Thus, while it is striking that the different degrees of treatment response among the four histological subtypes appear to reflect the order of aggressiveness of these lymphomas, the main finding on this point is that both glucose metabolism and cell density of the lesions show a marked response to their respective standard therapy regimens in all examined lymphoma subtypes as early as 48–72 h after treatment initiation.

Notably, in DLBCL, for which two different treatment regimens (R-CHOP and DA-EPOCH-R) were used, the observed differences between the two treatment groups point towards a stronger and/or more rapid effect of R-CHOP on glucose metabolism and diffusivity/cell density of the lymphomas in this early phase, which seems plausible because, with R-CHOP, both rituximab and the chemotherapeutic agents were administered on day 1, whereas with DA-EPOCH-R, rituximab was administered on day 1, and the chemotherapeutic agents were administered over 5 days. Thus, at the first PET/MR follow-up at 48–72 h after treatment initiation, patients in the DA-EPOCH-R arm had only received a fraction of the total chemotherapy dose, and lower peak levels. However, assignment to the two treatment groups was based on IPI score or the presence of double-hit lymphomas, i.e. only high-risk patients were chosen for treatment with DA-EPOCH-R, in accordance with previous studies [29, 30]. As a consequence, it is quite possible that the clinical entities in themselves are not comparable, and this led to, or at least contributed to, the observed differences in ultra-early responses on [^18^F]FDG PET/MR.

As mentioned above, DWI enables (indirect) assessment of cell density through assessment of tissue diffusivity, based on the underlying mechanism that in hypercellular tumours such as lymphoma, the extracellular space is compressed, limiting the local Brownian motion of water molecules. As a consequence, DWI may be used to capture treatment effects in terms of cell necrosis [31]. It is highly unlikely that, in our study, an isolated treatment-induced inflammatory fluid influx (which might have led to a T2-shine-through effect), rather than cell necrosis, was responsible for the observed ADC increase after treatment for several reasons. Firstly, a fluid influx in the absence of cell necrosis would lead to an increase in lesion size, which we did not observe. On the contrary, there was a small-to-moderate statistically significant decrease in morphological lesion volumes, which is indicative of cell necrosis. Secondly, a possible treatment-induced inflammatory reaction would not explain the obvious differences in ADC increase among the four histological lymphoma subtypes, as this would require a histology-dependent or treatment-dependent degree of inflammation for which there is presently no evidence; even more so as three of the lymphoma subgroups received rituximab-based treatment. Here, it is also important to note that treatment regimens for HL and DLBCL included the corticosteroid prednisone, which has been shown to lead to suppression of [^18^F]FDG uptake caused by the inflammatory influx early (i.e. on day 9) after cytotoxic treatment [32]. Thus, the decrease in [^18^F]FDG uptake early after treatment that was observed at the same time as the increase in diffusivity (i.e. ADCs), also supports our theory that DWI does indeed capture treatment-induced cell necrosis, rather than inflammatory changes. This is also in agreement with the results of Papaevangelou et al. [31], who observed significant ADC increases in the presence of necrosis in human colon carcinoma xenografts on days 3 and 5 after treatment with irinotecan.

Conversely, the decrease in FDG uptake observed at the ultra-early follow-up in our study was probably also caused, at least in part, by cell death, as indicated by the simultaneous increase in diffusivity and lesion shrinkage. Nevertheless, particularly in the context of HL with its pronounced hyperplastic lymphoid reaction, the anti-inflammatory and lympholytic effects of prednisone on [^18^F]FDG uptake and on lesion volume must be taken into account. Furthermore, a previous in vitro study in breast cancer cells established the concept of treatment-induced “stunning” (i.e. a discrepancy between FDG uptake and viable cell number) 24 h after doxorubicin treatment, and thus before the earliest follow-up at 48–72 h after treatment initiation in our study [33]. In the latter study, the stunning effect was explained by declines in GLUT-1 and HKII levels [33]. Similarly, in a murine lymphoma model, a reduction in FDG uptake 16 h after initiation of etoposide treatment was explained by a decrease in the plasma membrane presentation of GLUT-1 and GLUT-3 [23]. It is therefore difficult to judge whether, and to what degree, a possible stunning effect (in combination with the anti-inflammatory effect of prednisone in HL and DLBCL) may have contributed to the observed reduction in FDG uptake in our study, and whether it could partly explain the more pronounced changes in SUV and MTV, compared with the changes in ADC, especially since doxorubicin was also part of the DLBCL and HL treatment regimens in the present study. However, stunning would not have affected ADC and lesion volume changes, which showed the same general trends including differences in response among lymphoma subtypes. Thus, the observed reduction in FDG uptake after treatment initiation can at least not entirely be attributed to stunning and anti-inflammatory treatment effects. Only in three patients was an atypical behaviour observed (initial strong decreases in SUV and MTV from baseline to 48–72 h, followed by increases in SUV and MTV from 48–72 h to 1 week). Unfortunately, in these three patients, we were not able to distinguish between an initial stunning effect that was captured by [^18^F]FDG PET at 48–72 h and an inflammatory “flare” reaction that occurred between 48–72 h and 1 week, as this would have required histological evaluation.

Several studies have demonstrated the prognostic value of interim [^18^F]-FDG PET, typically performed after two to four therapy cycles, in different lymphoma subtypes, most convincingly in HL [14, 34]. For interim DWI, on the other hand, relatively few data are presently available with regard to its possible value for clinical outcome prediction. Based on a small series of 14 lymphoma patients (12 with DLBCL), De Paepe et al. found that changes in ADC between baseline and follow-up after 2 weeks (as well as 4 weeks) of treatment were significantly correlated with progression-free survival [35]. In a mixed population of 20 patients, 7 with HL and 13 with NHL including aggressive and indolent subtypes, Horger et al. found that changes in ADC after 1 week of treatment predicted outcome as assessed 6 months after the end of treatment [24]. Finally, in a series of 15 patients with FDG-avid MALT lymphoma, Mayerhoefer et al. found that changes in ADC between baseline and interim imaging differed significantly between patients with complete remission and those with residual disease at the end of treatment [10]. To our knowledge, no long-term data regarding the predictive value of therapy-induced ADC changes in lymphoma are presently available.

Our study had several limitations. No posttreatment histological data were available for comparison with the quantitative imaging parameters of the target lesions, because biopsies are rarely performed in routine clinical practice, and purely study-related biopsies were not performed because of ethical considerations. For calculation of MTVs, we used a 41% SUVmax cut-off, as previously recommended [26–28]. However, it is well-known that major differences in glucose metabolism (and the associated [^18^F]FDG uptake) exist between different lymphoma subtypes [36, 37]. We therefore cannot rule out the possibility that the use of a 41% SUVmax threshold led to a small-to-moderate MTV overestimation in patients with indolent lymphomas (i.e. FL, MCL) which frequently show less-pronounced FDG uptake.

In conclusion, our preliminary data clearly suggest that patients with HL and common NHL subtypes may show a substantial response to standard chemo- or immunochemotherapy as early as during the first 48–72 h after treatment initiation, which can be captured in vivo by [^18^F]FDG PET/MR. Treatment-induced changes in glucose metabolism appear to precede changes in diffusivity/cell density of lymphoma tissue, which in turn precede changes in lesion volume. Flare reactions that may be detrimental to [^18^F]FDG PET appear to be rare events during the investigated time period early after treatment initiation. The degree of treatment response in this very early treatment phase may be histology-dependent (with a trend for stronger responses in more aggressive lymphoma subtypes), but may also depend on the treatment regimen used. Notably, substantial differences exist between patients with regard to this very early treatment response, even between those with the same histological lymphoma subtype and treatment regimen. Consequently, follow-up is warranted to investigate the possible clinical significance of the observed changes in imaging parameters in terms of prognosis and survival.

## Electronic supplementary material

ESM 1 (PDF 891 kb)

ESM 2 (PDF 297 kb)

## References

[1] Cheson BD, Fisher RI, Barrington SF, Cavalli F, Schwartz LH, Zucca E (2014). Recommendations for initial evaluation, staging, and response assessment of Hodgkin and non-Hodgkin lymphoma: the Lugano classification. J Clin Oncol.

[2] Ghielmini M, Vitolo U, Kimby E, Montoto S, Walewski J, Pfreundschuh M (2013). ESMO guidelines consensus conference on malignant lymphoma 2011 part 1: diffuse large B-cell lymphoma (DLBCL), follicular lymphoma (FL) and chronic lymphocytic leukemia (CLL). Ann Oncol.

[3] Kostakoglu L, Cheson BD (2014). Current role of FDG PET/CT in lymphoma. Eur J Nucl Med Mol Imaging.

[4] Luminari S, Biasoli I, Arcaini L, Versari A, Rusconi C, Merli F (2013). The use of FDG-PET in the initial staging of 142 patients with follicular lymphoma: a retrospective study from the FOLL05 randomized trial of the Fondazione Italiana Linfomi. Ann Oncol.

[5] Cronin CG, Swords R, Truong MT, Viswanathan C, Rohren E, Giles FJ (2010). Clinical utility of PET/CT in lymphoma. AJR Am J Roentgenol.

[6] Watanabe R, Tomita N, Takeuchi K, Sakata S, Tateishi U, Tanaka M (2010). SUVmax in FDG-PET at the biopsy site correlates with the proliferation potential of tumor cells in non-Hodgkin lymphoma. Leuk Lymphoma.

[7] Lyng H, Haraldseth O, Rofstad EK (2000). Measurement of cell density and necrotic fraction in human melanoma xenografts by diffusion weighted magnetic resonance imaging. Magn Reson Med.

[8] Lin C, Luciani A, Itti E, Haioun C, Safar V, Meignan M (2012). Whole-body diffusion magnetic resonance imaging in the assessment of lymphoma. Cancer Imaging.

[9] Sun M, Cheng J, Zhang Y, Wang F, Meng Y, Fu X (2016). Application value of diffusion weighted whole body imaging with background body signal suppression in monitoring the response to treatment of bone marrow involvement in lymphoma. J Magn Reson Imaging.

[10] Mayerhoefer ME, Karanikas G, Kletter K, Kiesewetter B, Weber M, Rausch I (2016). Can interim 18F-FDG PET or diffusion-weighted MRI predict end-of-treatment outcome in FDG-avid MALT lymphoma after rituximab-based therapy? A preliminary study in 15 patients. Clin Nucl Med.

[11] Toledano-Massiah S, Luciani A, Itti E, Zerbib P, Vignaud A, Belhadj K (2015). Whole-body diffusion-weighted imaging in Hodgkin lymphoma and diffuse large B-cell lymphoma. Radiographics.

[12] Mayerhoefer ME, Karanikas G, Kletter K, Prosch H, Kiesewetter B, Skrabs C (2015). Evaluation of diffusion-weighted magnetic resonance imaging for follow-up and treatment response assessment of lymphoma: results of an 18F-FDG-PET/CT-controlled prospective study in 64 patients. Clin Cancer Res.

[13] Barrington SF, Mikhaeel NG, Kostakoglu L, Meignan M, Hutchings M, Müeller S (2014). Role of imaging in the staging and response assessment of lymphoma: consensus of the International Conference on Malignant Lymphomas Imaging Working Group. J Clin Oncol.

[14] Furth C, Steffen IG, Amthauer H, Ruf J, Misch D, Schönberger S (2009). Early and late therapy response assessment with [18F]fluorodeoxyglucose positron emission tomography in pediatric Hodgkin’s lymphoma: analysis of a prospective multicenter trial. J Clin Oncol.

[15] Itti E, Meignan M, Berriolo-Riedinger A, Biggi A, Cashen AF, Véra P (2013). An international confirmatory study of the prognostic value of early PET/CT in diffuse large B-cell lymphoma: comparison between Deauville criteria and ΔSUVmax. Eur J Nucl Med Mol Imaging.

[16] Casasnovas RO, Meignan M, Berriolo-Riedinger A, Bardet S, Julian A, Thieblemont C (2011). SUVmax reduction improves early prognosis value of interim positron emission tomography scans in diffuse large B-cell lymphoma. Blood.

[17] Borchmann P, Haverkamp H, Lohri A, Mey U, Kreissl S, Greil R (2017). Progression-free survival of early interim PET-positive patients with advanced stage Hodgkin’s lymphoma treated with BEACOPPescalated alone or in combination with rituximab (HD18): an open-label, international, randomised phase 3 study by the German Hodgkin Study Group. Lancet Oncol.

[18] Barrington SF, Kluge R (2017). FDG PET for therapy monitoring in Hodgkin and non-Hodgkin lymphomas. Eur J Nucl Med Mol Imaging.

[19] Lazarovici J, Terroir M, Arfi-Rouche J, Michot JM, Mussot S, Florea V (2017). Poor predictive value of positive interim FDG-PET/CT in primary mediastinal large B-cell lymphoma. Eur J Nucl Med Mol Imaging.

[20] Moon SH, Lee AY, Kim WS, Kim SJ, Cho YS, Choe YS (2017). Value of interim FDG PET/CT for predicting outcome of patients with angioimmunoblastic T-cell lymphoma. Leuk Lymphoma.

[21] Mikhaeel NG, Smith D, Dunn JT, Phillips M, Møller H, Fields PA (2016). Combination of baseline metabolic tumour volume and early response on PET/CT improves progression-free survival prediction in DLBCL. Eur J Nucl Med Mol Imaging.

[22] Schöder H, Zelenetz AD, Hamlin P, Gavane S, Horwitz S, Matasar M (2016). Prospective study of 3′-deoxy-3′-18F-fluorothymidine PET for early interim response assessment in advanced-stage B-cell lymphoma. J Nucl Med.

[23] Witney TH, Kettunen MI, Day SE, Hu DE, Neves AA, Gallagher FA (2009). A comparison between radiolabeled fluorodeoxyglucose uptake and hyperpolarized (13)C-labeled pyruvate utilization as methods for detecting tumor response to treatment. Neoplasia.

[24] Horger M, Claussen C, Kramer U, Fenchel M, Lichy M, Kaufmann S (2014). Very early indicators of response to systemic therapy in lymphoma patients based on alterations in water diffusivity – a preliminary experience in 20 patients undergoing whole-body diffusion-weighted imaging. Eur J Radiol.

[25] Hagtvedt T, Seierstad T, Lund KV, Løndalen AM, Bogsrud TV, Smith HJ (2015). Diffusion-weighted MRI compared to FDG PET/CT for assessment of early treatment response in lymphoma. Acta Radiol.

[26] Meignan M, Sasanelli M, Casasnovas RO, Luminari S, Fioroni F, Coriani C (2014). Metabolic tumour volumes measured at staging in lymphoma: methodological evaluation on phantom experiments and patients. Eur J Nucl Med Mol Imaging.

[27] Meignan M, Cottereau AS, Versari A, Chartier L, Dupuis J, Boussetta S (2016). Baseline metabolic tumor volume predicts outcome in high-tumor-burden follicular lymphoma: a pooled analysis of three multicenter studies. J Clin Oncol.

[28] Cottereau AS, Lanic H, Mareschal S, Meignan M, Vera P, Tilly H (2016). Molecular profile and FDG-PET/CT total metabolic tumor volume improve risk classification at diagnosis for patients with diffuse large B-cell lymphoma. Clin Cancer Res.

[29] Wilson WH, Dunleavy K, Pittaluga S, Hegde U, Grant N, Steinberg SM (2008). Phase II study of dose-adjusted EPOCH-rituximab in untreated diffuse large B-cell lymphoma with analysis of germinal center and post-germinal center biomarkers. J Clin Oncol.

[30] Howlett C, Snedecor SJ, Landsburg DJ, Svoboda J, Chong EA, Schuster SJ (2015). Front-line, dose-escalated immunochemotherapy is associated with a significant progression-free survival advantage in patients with double-hit lymphomas: a systematic review and meta-analysis. Br J Haematol.

[31] Papaevangelou E, Almeida GS, Jamin Y, Robinson SP, deSouza NM (2015). Diffusion-weighted MRI for imaging cell death after cytotoxic or apoptosis-inducing therapy. Br J Cancer.

[32] Brepoels L, Stroobants S, Vandenberghe P, Spaepen K, Dupont P, Nuyts J (2007). Effect of corticosteroids on 18F-FDG uptake in tumor lesions after chemotherapy. J Nucl Med.

[33] Engles JM, Quarless SA, Mambo E, Ishimori T, Cho SY, Wahl RL (2006). Stunning and its effect on 3H-FDG uptake and key gene expression in breast cancer cells undergoing chemotherapy. J Nucl Med.

[34] Zaucha JM, Malkowski B, Chauvie S, Subocz E, Tajer J, Kulikowski W (2017). The predictive role of interim PET after the first chemotherapy cycle and sequential evaluation of response to ABVD in Hodgkin’s lymphoma patients – the Polish Lymphoma Research Group (PLRG) Observational Study. Ann Oncol.

[35] De Paepe K, Bevernage C, De Keyzer F, Wolter P, Gheysens O, Janssens A (2013). Whole-body diffusion-weighted magnetic resonance imaging at 3 Tesla for early assessment of treatment response in non-Hodgkin lymphoma: a pilot study. Cancer Imaging.

[36] Paes FM, Kalkanis DG, Sideras PA, Serafini AN (2010). FDG PET/CT of extranodal involvement in non-Hodgkin lymphoma and Hodgkin disease. Radiographics.

[37] Weiler-Sagie M, Bushelev O, Epelbaum R, Dann EJ, Haim N, Avivi I (2010). (18)F-FDG avidity in lymphoma readdressed: a study of 766 patients. J Nucl Med.

