# Supplementary material for: Ultra-early response assessment in lymphoma treatment: [18F]FDG PET/MR captures changes in glucose metabolism and cell density within the first 72 hours of treatment
Source: Eur J Nucl Med Mol Imaging. 2018 Feb 26;45(6):931–40. doi: 10.1007/s00259-018-3937-z (PMC5915494; doi:10.1007/s00259-018-3937-z)

ONLINE RESOURCE 1

Manuscript title: Ultra-early Response Assessment in Lymphoma Treatment: [18F]FDG-PET/MR Captures Changes in Glucose Metabolism and Cell Density Within the First 72 Hours of Treatment

Journal: European Journal of Nuclear Medicine and Molecular Imaging

Authors: Marius E. Mayerhoefer, Markus Raderer, Ulrich Jaeger, Philipp Staber, Barbara Kiesewetter, Daniela Senn, Ferdia A. Gallagher, Kevin Brindle, Edit Porpaczy, Michael Weber, Dominik Berzaczy, Ingrid Simonitsch-Klupp, Christian Sillaber, Cathrin Skrabs, and Alexander Haug

Correspondence:

Marius E. Mayerhoefer, MD , PhD

Department of Biomedical Imaging and Image-guided Therapy

Medical University of Vienna, Austria

Waehringer Guertel 18-20, 1090 Vienna, Austria

Tel.: +43 1 40400 48180; Fax: +43 1 40400 48980

Email: marius.mayerhoefer@meduniwien.ac.at

Online Resource 1. Line graphs depicting the patient-based rates of change of standardized [18F]FDG uptake values (SUVmean), apparent diffusion coefficients (ADCmean), metabolic tumor volumes (MTV) and morphological tumor volumes (VOL) between the three time points, individually for each patient and lymphoma subtype. Considerable interindividual differences, in terms of treatment response, are visible for all lymphoma subtypes.

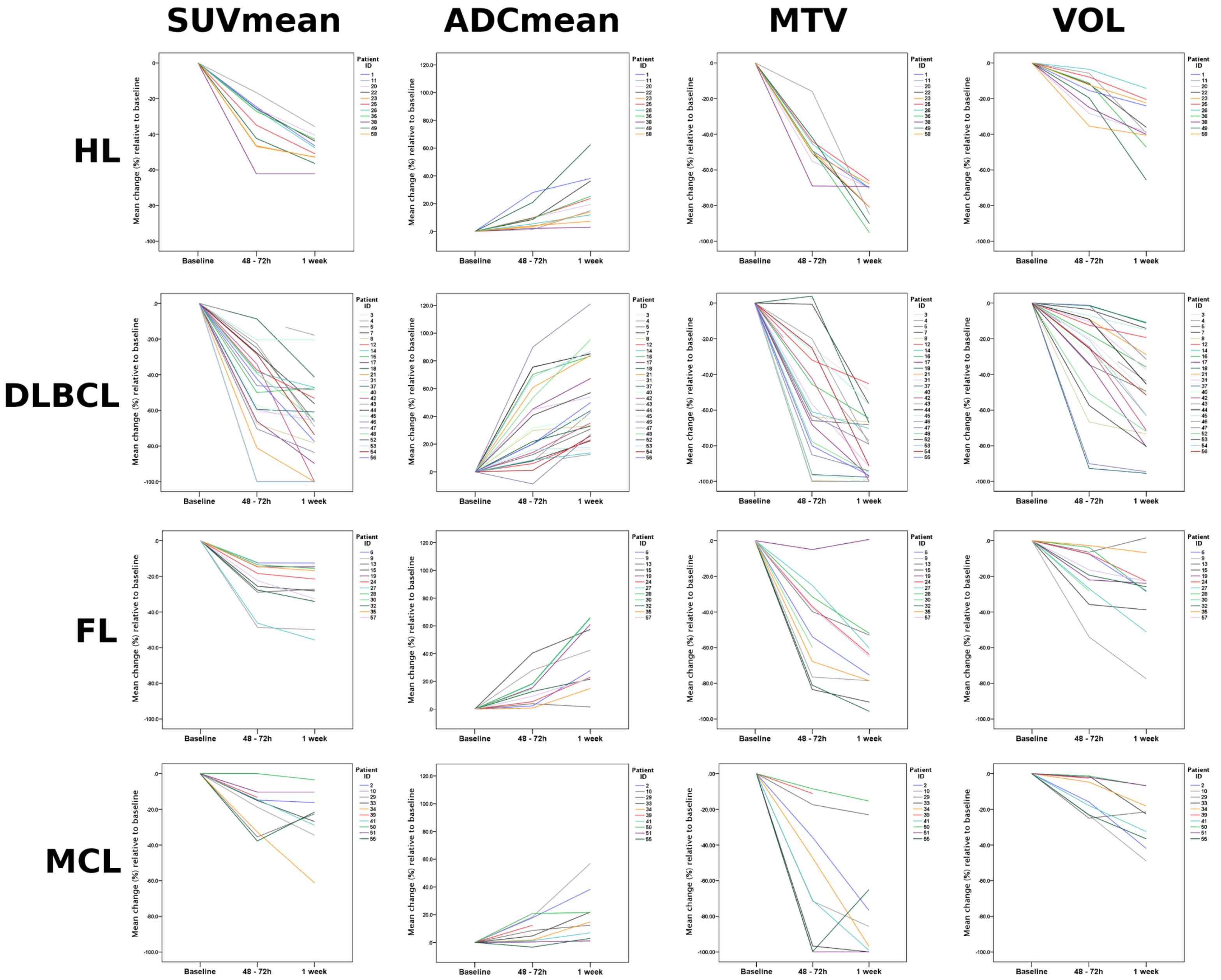

Supplement: Supplementary file 1 — (PDF 891 kb) [file 259_2018_3937_MOESM1_ESM.pdf]
