# Supplementary material for: Ultra-early response assessment in lymphoma treatment: [18F]FDG PET/MR captures changes in glucose metabolism and cell density within the first 72 hours of treatment
Source: Eur J Nucl Med Mol Imaging. 2018 Feb 26;45(6):931–40. doi: 10.1007/s00259-018-3937-z (PMC5915494; doi:10.1007/s00259-018-3937-z)

ONLINE RESOURCE 2

Manuscript title: Ultra-early Response Assessment in Lymphoma Treatment: [18F]FDG-PET/MR Captures Changes in Glucose Metabolism and Cell Density Within the First 72 Hours of Treatment

Journal: European Journal of Nuclear Medicine and Molecular Imaging

Authors: Marius E. Mayerhoefer, Markus Raderer, Ulrich Jaeger, Philipp Staber, Barbara Kiesewetter, Daniela Senn, Ferdia A. Gallagher, Kevin Brindle, Edit Porpaczy, Michael Weber, Dominik Berzaczy, Ingrid Simonitsch-Klupp, Christian Sillaber, Cathrin Skrabs, and Alexander Haug

Correspondence:

Marius E. Mayerhoefer, MD , PhD

Department of Biomedical Imaging and Image-guided Therapy

Medical University of Vienna, Austria

Waehringer Guertel 18-20, 1090 Vienna, Austria

Tel.: +43 1 40400 48180; Fax: +43 1 40400 48980

Email: marius.mayerhoefer@meduniwien.ac.at

Online Resource 2. Line graphs depicting the patient-based rates of change of standardized [18F]FDG uptake values (SUVmean) and apparent diffusion coefficients (ADCmean) in the two DLBCL groups between the three time points, individually for each patient and treatment regimen (R-CHOP or DA-EPOCH-R). There is a trend for a stronger effect of R-CHOP within the first 48-72 hours, compared to DA-EPOCH-R, even though both treatment groups show major interindividual differences in terms of response.

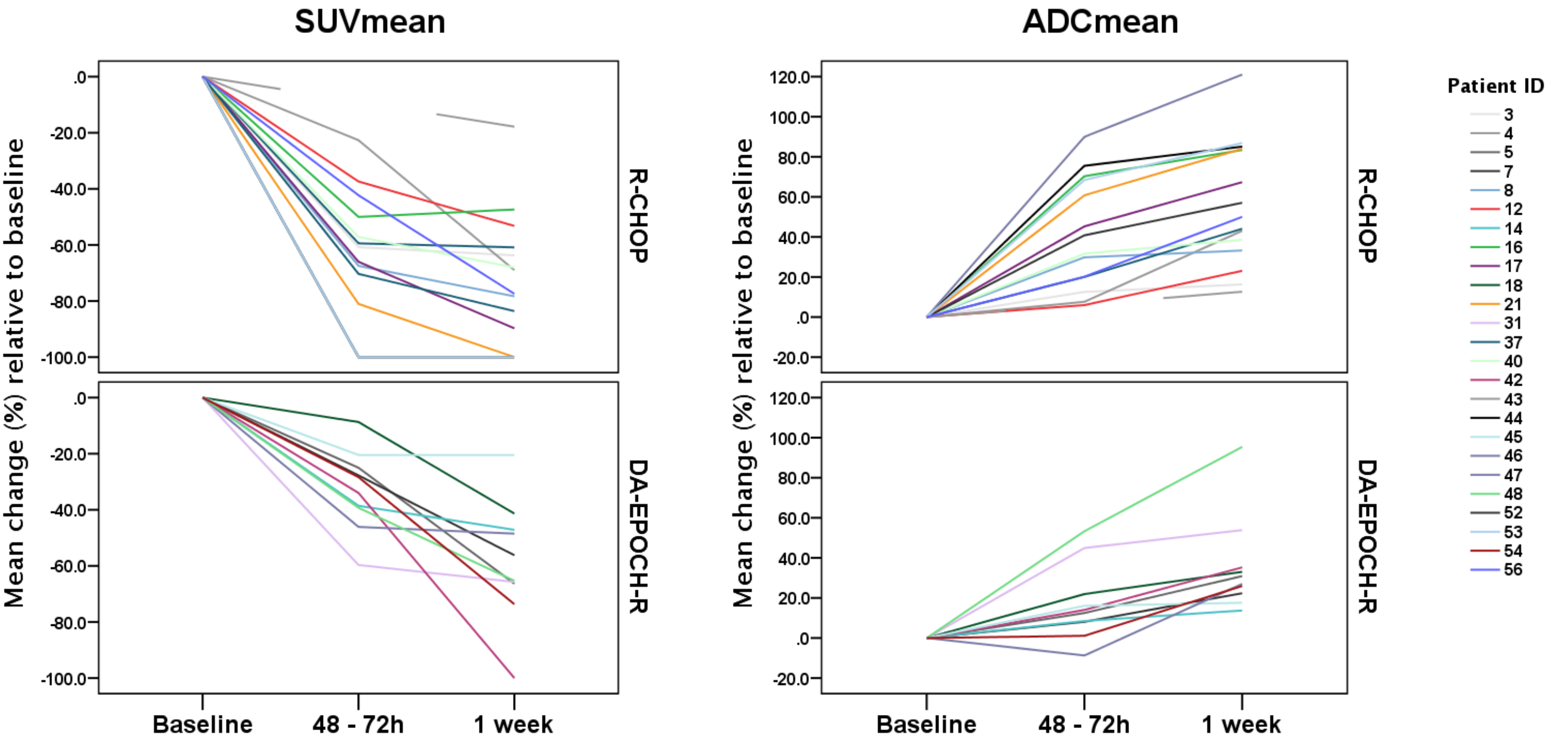

Supplement: Supplementary file 2 — (PDF 297 kb) [file 259_2018_3937_MOESM2_ESM.pdf]
